# HALP score and GNRI: Simple and easily accessible indexes for predicting prognosis in advanced stage NSCLC patients. The İzmir oncology group (IZOG) study

**DOI:** 10.3389/fnut.2022.905292

**Published:** 2022-08-18

**Authors:** Zeynep Gülsüm Güç, Ahmet Alacacıoğlu, Mehmet Eren Kalender, Utku Oflazoğlu, Sinan Ünal, Yaşar Yıldız, Tarık Salman, Yüksel Küçükzeybek, Mustafa Oktay Tarhan

**Affiliations:** ^1^Department of Medical Oncology, İzmir Katip Çelebi University, Atatürk Training and Research Hospital, İzmir, Turkey; ^2^Department of Medical Oncology, Institute of Oncology, Dokuz Eylül University, İzmir, Turkey

**Keywords:** geriatric nutritional risk index (GNRI), non-small cell lung cancer, hemoglobin-albumin-lymphocyte-platelet score, prognostic, metastatic

## Abstract

**Objective:**

The Hemoglobin, Albumin, Lymphocyte, and Platelet (HALP) Score and the Geriatric Nutrition Risk Index (GNRI) are used as prognostic factors in different types of cancers. In this study we analyzed the prognostic value of the HALP Score and the GNRI calculated prior to first-line treatment in patients diagnosed with *de novo* metastatic non-small cell lung cancer (mNSCLC).

**Materials and methods:**

*De novo* mNSCLC patients were retrospectively evaluated from January 2016 to December 2019. Patients with Driver’s mutation, severe comorbidities, active infection, or insufficient organ function, and those receiving anti-inflammatory treatment were excluded from the study. Optimal cut-off points for the HALP score and the GNRI were calculated with the receiver operating characteristic (ROC) curve analysis. Predictive factors for overall survival (OS) were assessed with univariate and multivariate Cox proportional hazard analyses, and OS was studied with the Kaplan–Meier analysis.

**Results:**

The study included 401 patients in total. In the ROC curve analysis, the cut-off points were found 23.24 (AUC = 0.928; 95% CI: 0.901–0.955, *p* < 0.001) for HALP, and 53.60 (AUC = 0.932; 95% CI: 0.908–0.955, *p* < 0.001) for GNRI. Groups with lower HALP scores and lower GNRI had significantly shorter OS compared to those with higher HALP scores and GNRIs. Univariate analysis showed that male gender, smoking, high ECOG score, low HALP score and low GNRI were associated with worse survival rates. Multivariate analysis showed that low HALP score (HR = 2.988, 95% CI: 2.065–4.324, *p* < 0.001); low GNRI score (HR = 2.901, 95% CI: 2.045–4.114, *p* < 0.001) and smoking history (HR = 1.447, 95% CI: 1.046–2.001, *p* = 0.025) were independent factors associated with worse OS rates.

**Conclusion:**

Our study showed the HALP score and the GNRI to be of prognostic value as simple, cost-effective, and useful markers that predict OS in *de novo* mNSCLC patients.

## Introduction

Lung cancers are among the top causes of cancer-related deaths in many industrialized countries, in women and men alike, accounting for about one-fifth of all cancer-related deaths (1). Adenocarcinoma and squamous cell lung cancers are the most common subtypes of non-small cell lung cancer (NSCLC) and account for 85% of lung cancers. Unfortunately, only 16% of the patients present with localized disease at the time of initial diagnosis (2). Many patients already have reached an advanced stage by this time and miss the opportunity for early treatment (3). Five-year survival rate is 67% in T1N0 patients, 23% in T1-3N2 patients, and 1–10% in metastatic patients. Individualized treatments were developed in mNSCLC by identifying Driver’s mutations and molecular features, and significant increases in life expectancy were achieved with the application of these treatments (4).

There is an increasing focus on identifying the molecular changes critical to the oncogenic phenotype of NSCLC and the treatments targeting Driver’s mutations. Moreover, the use of immunomodulatory drugs, especially of anti-PD1 drugs in advanced NSCLC patients is increasing. Yet, majority of the patients are still treated with cytotoxic chemotherapy for reasons such as their molecular/pathological characteristics or the unavailability of other treatment options. Availability of prognostic and predictive indexes that allow physicians to make the most suitable treatment decision for these patients are of great importance.

Current studies show that the systemic inflammatory response is associated with tumor characteristics such as proliferation, invasion, metastasis, and that inflammation has a significant role in the formation and growth of tumors (5, 6). Blood cells affect tumor cells through adaptive immune response by secreting different cytokines that help various inflammatory processes (7). The neutrophil-to-lymphocyte ratio, the platelet-lymphocyte ratio, the lymphocyte-monocyte ratio, and inflammatory indexes such as the prognostic nutritional index have been used to predict prognosis in different cancer types (8, 9).

It is known that a combination of these parameters can predict the patient’s prognosis much better than a single index. To that end, the HALP score—an index which is calculated based on hemoglobin, albumin, lymphocyte, and platelet levels—has been recently defined. The HALP score assesses both the immune system and the nutritional status of the patient. The score has been reported to be a good prognostic marker in various types of cancers, including gastrointestinal and genitourinary (10, 11). These parameters can be calculated simply based on the laboratory parameters of patients used in everyday practice.

Malnutrition is often seen in oncology patients. The prevalence of malnutrition and weight loss among cancer patients ranges from 20% to 70% depending on the type and stage of the cancer, and patients with upper gastrointestinal tract, lung and pancreatic cancer are at greater risk. Many patients suffer nutritional deficiency even at the diagnosis stage (12). The Geriatric Nutrition Risk Index (GNRI) was first defined in 2005 by Bouillanne et al. (13) and is a combination of the serum albumin level and the ratio of body weight to ideal body weight.

Although a number of clinicopathological indicators such as the metastasis site, histology, PD-L1 expression, tumor mutation burden, and EGFR, ALK, ROS, KRAS mutation may predict the prognosis of NSCLC, simpler and more precise indexes are also needed. In this study, we aimed to investigate whether pre-treatment HALP score and GNRI could serve as independent and strong risk factors of overall survival (OS) in mNSCLC patients.

## Materials and methods

### Data collection and follow-up

**FIGURE 1:**
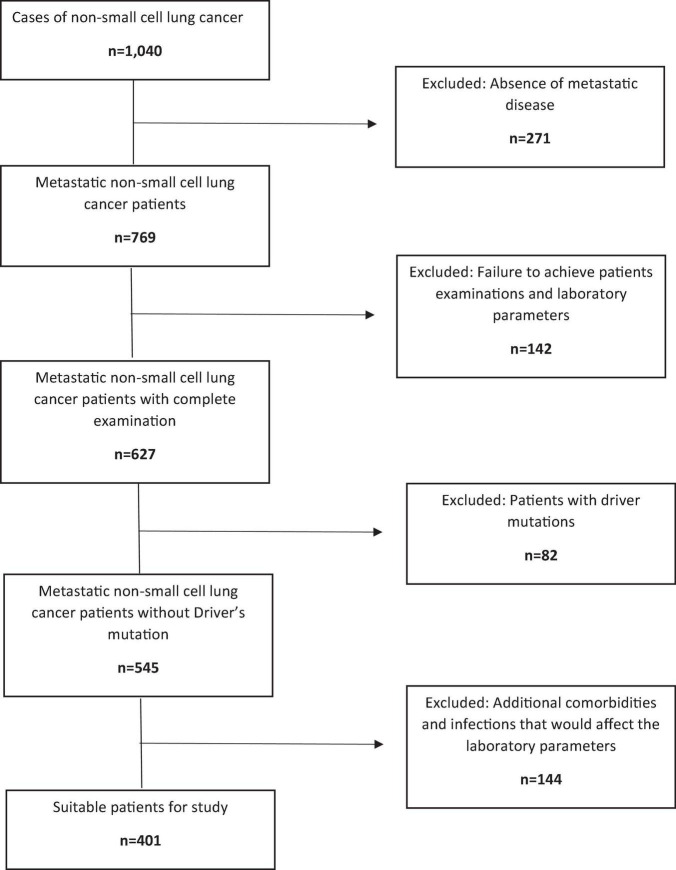
Flow chart showing the patient selection process.

The method and the procedure of the study were approved by the Ethics Committee of the University. Records of 1,040 patients diagnosed with NSCLC were retrospectively reviewed. Patients who received targeted therapy or were Driver’s mutation carriers were excluded since these could further affect prognosis. Also, patients with severe comorbidities, active infection or inadequate organ function and those receiving anti-inflammatory therapy were excluded since these conditions might affect inflammatory response. Patients were also excluded if their data needed to calculate their prognostic indexes were not available in the records. As a result, 401 *de novo* mNSCLC patients who were treated and followed-up in our medical oncology clinic from January 2016 to December 2019 were included (Figure 1). Patients’ age, demographic data, weight, height, body mass index (BMI), comorbidities, ECOG (Eastern Cooperative Oncology Group) status, staging at diagnosis, histological type of the tumor, and chemotherapy regimen used, were recorded. Lymphocyte and platelet counts, and serum albumin levels that were examined 1 week before the first cycle of chemotherapy were retrieved from the laboratory information system for the calculation of the HALP score and the GNRI.

The HALP score was calculated according to the following formula: hemoglobin (g/L) × albumin (g/L) × lymphocytes (/L)/platelets (/L). GNRI was calculated using serum albumin concentration and body weight as described elsewhere (14) with the following formula: 14.87 × serum albumin concentration (g/l) + 41.7 × weight/ideal weight (kg); with ideal body weight calculated as: 22 × square of height (m).

### Statistical analyses

The Statistical Package for Social Sciences for Windows 20.0 (SPSS, Inc., Chicago, IL, United States) was used for analysis. Overall Survival (OS) was defined as the time from diagnosis to death or the last visit. Descriptive statistics summarized frequencies and percentages for categorical, mean, and standard deviation for continuous variables. Categorical variables were compared with the Independent Samples *T*-test and categorical parameters with the χ2 test. The power of the HALP scores and the GNRI were analyzed using ROC curve analysis. A significant cut-off point was observed, and sensitivity, specificity, and positive and negative predictive values were detected. Survival analyses of prognostic indexes, and clinical and pathological features were calculated using the Kaplan–Meier method (log-rank test). Multivariate analyses were used to identify independent prognostic variables based on a stepwise Cox proportional hazards regression model and variables that potentially affected survival (*p* < 0.05 in univariate analyses). Parameters that appeared significant in univariate analysis for survival and did not show multicollinearity were included in the Cox multivariate regression analysis. The 95% confidence interval (CI) was used to indicate the relationship between survival time and each independent factor. Statistical significance level was *p* < 0.05.

## Results

Clinical characteristics of the 401 patients included in the study were analyzed: their mean age was 63.47 ± 9.75 years (median, 63 years); 211 patients were < 65 years and 190 patients were ≥ 65 years of age; 317 (79.1%) of the patients were male. The most common histopathological type was adenocarcinoma at a rate of 51.1% (205 patients). Mean follow-up time was 18 (range, 1–80) months. Two-hundred-and-sixty-one patients (65.1%) died by the end of the follow-up period. All patients received platinum-based combination therapy as first-line chemotherapy. The most commonly used regime was the cisplatin-gemcitabine combination (204 patients, 50.9%), followed by the carboplatin-paclitaxel combination (170 patients, 42.4%) and the carboplatin-gemcitabine combination (27 patients, 6.7%). Two-hundred-and-forty-two patients (60.3%) were progressive after their first-line treatment. Basic characteristics of the patients are given in Table 1.

**TABLE 1:** Clinicopathologic characteristics of the whole cohort according to HALP and GNRI.

Parameters	Number of patients (%)	HALP score			GNRI		
		High (n)	Low (n)	*p*	High (n)	Low (n)	*p*
**Age**							
< 65	211 (52.6%)	98	113	0.064	104	107	
≥ 65	190 (47.4%)	73	117		75	115	**0.030**
**Gender**							
Female	84 (20.9%)	45	39	**0.016**	55	29	
Male	317 (79.1%)	126	191		124	193	**< 0.001**
**Smoking history**							
No	91 (22.7%)	45	46	0.085	56	35	
Yes	310 (77.3%)	126	184		123	187	**< 0.001**
**Histology**							
Adenocarcinoma	205 (51.1%)	90	115	0.411	88	117	
Squamous cell carcinoma	135 (33.7%)	53	82		58	77	
Large cell carcinoma	34 (8.4%)	13	21		15	19	0.127
Other	27 (6.8%)	15	12		18	9	
**ECOG**							
ECOG 0–1	228 (56.9%)	155	73	** < 0.001**	140	88	**< 0.001**
ECOG 2–3	173 (43.1%)	16	157		39	134	
**BMI (kg/m2)**							
BMI < 24	20 (5%)	8	12	0.499	6	14	
BMI ≥ 24	381 (95%)	163	218		173	208	0.131
**Chemotherapy drug combination** Cisplatin-gemcitabine	204 (50.9%)	95	109	0.219	104	100	
Carboplatin-paclitaxel	170 (42.4%)	64	106		65	105	**0.034**
Carboplatin-gemcitabine	27 (6.7%)	12	15		10	17	
**Treatment Response**							
Stable/responsive	159 (39.7%)	123	36	** < 0.001**	129	30	
Progress	242 (60.3%)	48	194		50	192	**< 0.001**

BMI, Body mass index; ECOG, Eastern Cooperative Oncology Group; HALP, Hemoglobin, albumin, lymphocyte, and platelet; GNRI, Geriatric Nutritional Risk Index. Bold values indicate the statistically significant.

**FIGURE 2:**
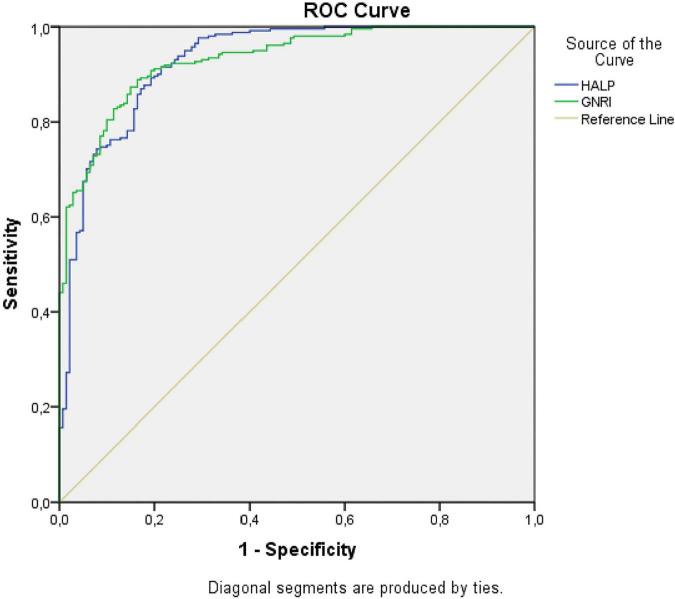
ROC curve for HALP score and GNRI. HALP, Hemoglobin, albumin, lymphocyte, and platelet; GNRI, Geriatric Nutritional Risk Index.

The patients had a median HALP score of 29.08 (range, 1.95–146.23) and a median GNRI of 54.26 (range, 19.17–121.66). ROC analysis was done to determine the most appropriate cut-off points for the HALP score and the GNRI levels. The ROC curve showed an optimal cut-off point of 23.24 (AUC = 0.928; 95% CI: 0.901–0.955, *p* < 0.001) for the HALP score. For GNRI, the ROC curve showed an optimal cut-off point of 53.60 (AUC = 0.932; 95% CI: 0.908–0.955, *p* < 0.001) (Figure 2). Patients were divided into low and high HALP and GNRI groups according to these cut-off points.

The chi-squared test demonstrated the difference between the pre-treatment HALP score and the clinical characteristics. Male gender, high ECOG score and progressive disease after first-line platinum-based chemotherapy was found to be associated with a low HALP score. The HALP score demonstrated no differences based on age, history of smoking, BMI, tumor histology, and chemotherapy regimen. Analysis of the GNRI groups showed male gender, an ECOG performance score of 2–3, history of smoking, advanced age, chemotherapy regimen and progression after first-line treatment to be statistically significantly associated with low GNRI (*p* < 0.005) (Table 1).

**FIGURE 3:**
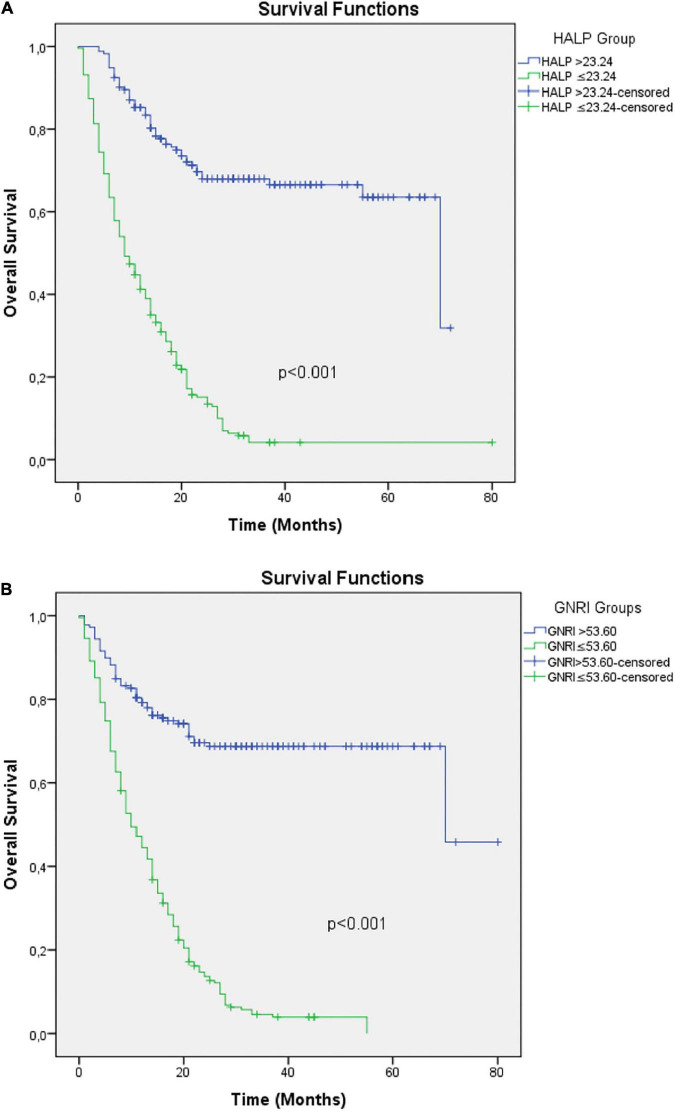
Kaplan–Meier curves for OS in patients with mNSCLC according to **(A)** HALP and **(B)** GNRI. HALP, Hemoglobin, albumin, lymphocyte, and platelet; GNRI, Geriatric Nutritional Risk Index.

Kaplan–Meier curves and log-rank test revealed that low HALP score and low GNRI were significantly associated with worse OS (all *p* < 0.001) (Figure 3).

In all 401 patients, univariate analysis identified male gender, smoking, high ECOG score, low HALP score and low GNRI score to be significantly associated with worse survival rates.

Eight variables, namely, age, gender, smoking history, ECOG performance score, histopathology, chemotherapy, HALP score and GNRI, were included in the multivariate Cox regression analysis. Independent factors that were associated with worse OS rates were low HALP score (HR = 2.988, 95% CI: 2.065–4.324, *p* ≤ 0.001); low GNRI score (HR = 2.901, 95% CI: 2.045–4.114, *p* ≤ 0.001) and smoking history (HR = 1.447, 95% CI: 1.046–2.001, *p* = 0.025) (Table 2).

**TABLE 2:** Univariate and multivariate analyses of prognostic factors for OS.

	Univariate analysis			Multivariate analysis		
	HR	95% CI	*P-value*	HR	95% CI	*P-value*
**Gender**						
Male	1.418	1.015–1.981	**0.040**	**–**	**–**	**–**
Female						
**Age**						
< 65	1,414	1.012–1.976	**0.042**	–	–	–
≥ 65						
**ECOG**						
0–1	1.388	1.032–1.867	**0.030**	**–**	**–**	**–**
2–3						
**Smoking**						
No	1.426	0.1.019–1.996	**0.038**	1.447	1.046–2.001	**0.025**
Yes						
**BMI**						
< 24	1.029	0.581–1.822	0.923			
≥ 24						
**Histology**						
Adenocarcinoma	1.424	1.019–1.991	**0.039**	–	–	–
Squamous cell carcinoma						
Large cell carcinoma						
Other						
**Chemotherapy drug combination**						
Cisplatin-gemcitabine	1.122	0.682–1.846	0.649	–	–	–
Carboplatin-paclitaxel						
Carboplatin-gemcitabine						
**HALP**						
Low	3.004	2.075–4.348	** < 0.001**	2.988	2.065–4.324	** < 0.001**
High						
**GNRI**						
Low	2.863	2.014–4.069	** < 0.001**	2.901	2.045–4.114	** < 0.001**
High						

OS, Overall survival; BMI, Body mass index; ECOG, Eastern Cooperative Oncology Group; HR, Hazard ratio; CI, Confidence interval; HALP, Hemoglobin, albumin, lymphocyte, and platelet; GNRI, Geriatric Nutritional Risk Index. Bold values indicate the statistically significant.

## Discussion

The presented study aimed to identify the value of pre-treatment HALP scores and GNRI levels in predicting OS in mNSCLC. Review of the literature showed our study to be the first to demonstrate in multivariate analysis that low HALP score (≤ 23.24) and low GNRI are prognostic markers that independently define worse OS. We assessed simple prognostic markers like complete blood count, serum albumin levels, height, and weight, which are based on basic laboratory and anthropometric measurements as part of the routine evaluation process for each patient.

Despite the emergence of new treatments, mNSCLC remains an incurable disease with significant morbidity and high symptom burden. Although some patients show improvement with anticancer therapy, in most patients survival is only a few months. A realistic understanding of its prognosis and the goals of cancer treatment will determine the treatment decision for both patients and physicians. The importance of our study comes forth at this point. The study cohort consisted of mNSCLC patients, excluding those who had Driver’s mutation—because of better prognosis and more treatment options available—or were treated with molecular target agents. After other characteristics that could potentially have confounding effects on prognosis were excluded per the relevant exclusion criteria of the study design, a total of 401 patients who were comparable in terms of clinicopathological characteristics and treatments were analyzed.

As consistent with the literature, the majority of the patients in our study were male with adenocarcinoma morphology (4). Since patient characteristics were standardized and organ functions, performance statuses and comorbidities were carefully reviewed, all patients, similarly, received platinum-based combination therapy as first-line treatment. No effect of treatment choice was observed on survival in univariate or multivariate analysis.

The roles of inflammatory response and nutritional status in predicting prognosis in cancer patients are being investigated with increasing interest (15–18). Serum albumin, a negative acute-phase marker, is synthesized by the liver and used to assess nutritional status. While hypoalbuminemia can be caused by malnutrition or hypercatabolism, it can also be due to systemic inflammation and increased cytokine release, which can lead to a weak immune response to cancer cells (19). Many studies have reported that poor survival outcomes were associated with hypoalbuminemia in different types of cancers (20, 21).

Lymphocytes are of critical importance in the host’s anti-cancer defense. Lymphocytes, which can release cytokines such as interferon−γ and tumor necrosis factor−alpha (TNF−α), can improve the prognosis by causing apoptosis, suppressing cancer cell proliferation, invasion, and migration (22, 23). As a result, lymphocytopenia can contribute to tumor growth.

That hemoglobin levels are directly associated with both survival and tumor development in cancer patients has been shown in several studies (24–26). Platelet stimulation is linked to metastasis, and platelets can also protect cancer cells from immune attack (27). Jurasz et al. showed that platelets inhibit tumor necrosis *via* TNF−α (28).

Basing on these data, it can be said that the HALP score, which is calculated using hemoglobin, lymphocytes, platelets, and albumin values, is a comprehensive index that measures the nutritional status and the immune health of patients. It has been shown to have a prognostic effect in gastric cancer (29), squamous cell carcinoma of the esophagus (30), colorectal cancer (10), renal cell carcinoma (31), bladder cancer (11) and small cell lung cancer (32). However, to our knowledge, there are no previously reported studies on the prognostic significance of the HALP score in patients with mNSCLC. This study showed a HALP score > 23.24 to be an independent prognostic factor in patients with *de novo* m NSCLC, and that improvements in the HALP score could, in turn, significantly improve OS in NSCLC patients. Another important point is that this relationship was shown by carefully excluding the variables such as active infection, organ dysfunction, use of anti-inflammatory drugs and serious comorbidities that could affect the results.

Malnutrition is commonly seen in oncology patients and the etiology of cancer-related malnutrition is multifactorial. Different methods have been validated for the assessment of nutritional status in cancer patients, but the gold standard has not yet been established. It is often difficult to evaluate the nutritional status of oncological patients, especially in clinical settings where time is limited, and complex nutritional assessment methods cannot be applied.

GNRI reflects the patient’s nutritional status as it is based on serum albumin concentration and BMI. Low GNRI scores are highly associated with malnutrition. GNRI is an objective and simple screening tool that stands out as a powerful prognostic factor in certain malignancies, but BMI and serum albumin levels by themselves are not strongly associated with the prognosis of cancer patients (33). Serum albumin is a simple and valuable marker that can reflect malnutrition or cachexia in cancer patients. Several studies have reported weight loss to be a poor prognostic factor in lung cancer patients (34, 35). Moreover, GNRI, which is calculated with a combination of these parameters, has been reported to be a useful prognostic factor in esophageal cancer and renal cell carcinoma (36, 37). In their study, Peng et al. demonstrated the prognostic importance of GNRI in terms of survival in mNSCLC patients (38). In a study conducted with 160 NSCLC patients, low GNRI was found to be associated with worse OS (39).

The results of our study showed that GNRI scores greater than 53.60 were associated with longer survival and revealed GNRI to be an independent marker predicting survival. While, however, our results are consistent with those reported in the literature, our study is the first to show these results in a larger and more standardized (without Driver’s mutation and variables affecting inflammatory response) patient group.

Smoking tobacco is known to be the main risk factor for lung cancer and associated with high morbidity and mortality. However, smoking is associated with better survival in NSCLC patients receiving immune checkpoint inhibitors in different lines of treatment (40). Our study, however, included only those patients that received chemotherapy, and smoking was defined as an independent factor associated with poor survival.

Whilst being the first to demonstrate the HALP score and the GNRI score as independent predictive factors for OS in *de novo* mNSCLC patients, its retrospective single-center design has been a limiting factor of our study. Nevertheless, the exclusion of patients with Driver’s mutation and those who received targeted therapy has, we believe, helped to eliminate any effects on prognosis, hence, to achieve reliable results. Our low/high HALP score and low/high GNRI groups had similar clinicopathological characteristics. Another limiting factor was the lack of consensus on the cut-off points for the HALP and GNRI scores in NSCLC due to the limited number of studies. A better understanding of the effects of the dynamic changes in HALP and GNRI scores on long-term survival will help to better understand the relationship between these scores and NSCLC. We hope that our study will provide a basis for further prospective multi-center studies that are needed to more clearly assess how these scores should be used in the follow-up and for the survival benefit of patients.

To conclude, the HALP score and the GNRI are reliable, simple, easily accessible, and inexpensive indexes for predicting the prognosis of advanced stage NSCLC patients treated with platinum-based chemotherapeutics. Low HALP score and GNRI level can be considered clinical triggers for nutritional support in patients with advanced NSCLC.

## Data availability statement

The raw data supporting the conclusions of this article will be made available by the authors, without undue reservation.

## Ethics statement

Written informed consent was obtained from the individual(s) for the publication of any potentially identifiable images or data included in this article.

## Author contributions

ZG and AA: study concept, study design, and statistical analysis. ZG and MK: data acquisition. ZG, SÜ, and YY: quality control of data. ZG and UO: data analysis and interpretation. ZG, TS, and AA: manuscript editing. ZG, YK, AA, and MT: manuscript review. All authors contributed to the article and approved the submitted version.

## References

[1] Bray F Ferlay J Soerjomataram I Siegel RL Torre LA Jemal A. Global cancer statistics 2018: GLOBOCAN estimates of incidence and mortality worldwide for 36 cancers in 185 countries. *CA Cancer J Clin.* (2018) 68:394–424. 10.3322/caac.21492 30207593

[2] Pisters KM Evans WK Azzoli CG Kris MG Smith CA Desch CE Cancer Care Ontario and American Society of Clinical Oncology adjuvant chemotherapy and adjuvant radiation therapy for stages I-IIIA resectable non-small-cell lung cancer guideline. *J Clin Oncol.* (2007) 25:5506–18. 10.1200/JCO.2007.14.1226 17954710

[3] Miller KD Nogueira L Mariotto AB Rowland JH Yabroff KR Alfano CM Cancer treatment and survivorship statistics, 2019. *CA Cancer J Clin.* (2019) 69:363–85. 10.3322/caac.21565 31184787

[4] Vickers AD Winfree KB Cuyun Carter G Kiiskinen U Jen MH Stull D Relative efficacy of interventions in the treatment of second-line non-small cell lung cancer: a systematic review and network meta-analysis. *BMC Cancer.* (2019) 19:353. 10.1186/s12885-019-5569-5 30987609 PMC6466705

[5] McMillan DC. Systemic inflammation, nutritional status and survival in patients with cancer. *Curr Opin Clin Nutr Metab Care.* (2009) 12:223–6. 10.1097/MCO.0b013e32832a7902 19318937

[6] Colotta F Allavena P Sica A Garlanda C Mantovani A. Cancer-related inflammation, the seventh hallmark of cancer: links to genetic instability. *Carcinogenesis.* (2009) 30:1073–81. 10.1093/carcin/bgp127 19468060

[7] Varga G Foell D. Anti-inflammatory monocytes-interplay of innate and adaptive immunity. *Mol Cell Pediatr.* (2018) 5:5. 10.1186/s40348-018-0083-4 29616417 PMC5882470

[8] Hong YF Chen ZH Wei L Ma XK Li X Wen JY Identification of the prognostic value of lymphocyte-to-monocyte ratio in patients with HBV-associated advanced hepatocellular carcinoma. *Oncol Lett.* (2017) 14:2089–96. 10.3892/ol.2017.6420 28789436 PMC5530031

[9] Magdy M Hussein T Ezzat A Gaballah A. Pre-treatment peripheral neutrophil-lymphocyte ratio as a prognostic marker in gastric cancer. *J Gastrointest Cancer.* (2019) 50:763–8. 10.1007/s12029-018-0144-x 30058031

[10] Jiang H Li H Li A Tang E Xu D Chen Y Preoperative combined hemoglobin, albumin, lymphocyte and platelet levels predict survival in patients with locally advanced colorectal cancer. *Oncotarget.* (2016) 7:72076–83. 10.18632/oncotarget.12271 27765916 PMC5342146

[11] Peng D Zhang CJ Gong YQ Hao H Guan B Li XS Prognostic significance of HALP (hemoglobin, albumin, lymphocyte and platelet in patients with bladder cancer after radical cystectomy. *Sci Rep.* (2018) 8:794. 10.1038/s41598-018-19146-y 29335609 PMC5768698

[12] Halpern-Silveira D Susin LR Borges LR Paiva SI Assunção MC Gonzalez MC. Body weight and fat-free mass changes in a cohort of patients receiving chemotherapy. *Support Care Cancer.* (2010) 18:617–25. 10.1007/s00520-009-0703-6 19621246

[13] Bouillanne O Morineau G Dupont C Coulombel I Vincent J-P Nicolis I Geriatric nutritional risk index: a new index for evaluating at-risk elderly medical patients. *Am J Clin Nutr.* (2005) 82:777–83. 10.1093/ajcn/82.4.777 16210706

[14] Hasselmann M Alix E. Tools and procedures for screening for malnutrition and its associated in risks in hospital. *Nutr Clin Metab.* (2003) 17:218–26.

[15] Rock CL Doyle C Demark-Wahnefried W Meyerhardt J Courneya KS Schwartz AL Nutrition and physical activity guidelines for cancer survivors. *CA Cancer J Clin.* (2012) 62:243–74. 10.3322/caac.21142 22539238

[16] Wang X Han H Duan Q Khan U Hu Y Yao X. Changes of serum albumin level and systemic inflammatory response in inoperable non-small cell lung cancer patients after chemotherapy. *J Cancer Res Ther.* (2014) 10:1019–23. 10.4103/0973-1482.137953 25579547

[17] Efremova M Rieder D Klepsch V Charoentong P Finotello F Hackl H Targeting immune checkpoints potentiates immunoediting and changes the dynamics of tumor evolution. *Nat Commun.* (2018) 9:32. 10.1038/s41467-017-02424-0 29296022 PMC5750210

[18] Lu H Ouyang W Huang C. Inflammation, a key event in cancer development. *Mol Cancer Res.* (2006) 4:221–33. 10.1158/1541-7786.MCR-05-0261 16603636

[19] Lucijanic M Veletic I Rahelic D Pejsa V Cicic D Skelin M Assessing serum albumin concentration, lymphocyte count and prognostic nutritional index might improve prognosis in patients with myelofibrosis. *Wien Klin Wochenschr.* (2018) 130:126–33. 10.1007/s00508-018-1318-z 29372410 PMC11136504

[20] Liu X Meng QH Ye Y Hildebrandt MA Gu J Wu X. Prognostic significance of pretreatment serum levels of albumin, LDH and total bilirubin in patients with non-metastatic breast cancer. *Carcinogenesis.* (2015) 36:243–8. 10.1093/carcin/bgu247 25524924

[21] Oñate-Ocaña LF Aiello-Crocifoglio V Gallardo-Rincón D Herrera-Goepfert R Brom-Valladares R Carrillo JF Serum albumin as a significant prognostic factor for patients with gastric carcinoma. *Ann Surg Oncol.* (2007) 14:381–9. 10.1245/s10434-006-9093-x 17160496

[22] Lim JA Oh CS Yoon TG Lee JY Lee SH Yoo YB The effect of propofol and sevoflurane on cancer cell, natural killer cell, and cytotoxic T lymphocyte function in patients undergoing breast cancer surgery: an in vitro analysis. *BMC Cancer.* (2018) 18:159. 10.1186/s12885-018-4064-8 29415668 PMC5803927

[23] Mantovani A Allavena P Sica A Balkwill F. Cancer-related inflammation. *Nature.* (2008) 454:436–44. 10.1038/nature07205 18650914

[24] Belcher DA Ju JA Baek JH Yalamanoglu A Buehler PW Gilkes DM The quaternary state of polymerized human hemoglobin regulates oxygenation of breast cancer solid tumors: a theoretical and experimental study. *PLoS One.* (2018) 13:e0191275. 10.1371/journal.pone.0191275 29414985 PMC5802857

[25] Caro JJ Salas M Ward A Goss G. Anemia as an independent prognostic factor for survival in patients with cancer: a systematic, quantitative review. *Cancer.* (2001) 91:2214–21. 10.1002/1097-0142(20010615)91:12<2214::AID-CNCR1251>3.0.CO;2-P 11413508

[26] Xia L Hu G Guzzo TJ. Prognostic significance of preoperative anemia in patients undergoing surgery for renal cell carcinoma: a meta-analysis. *Anticancer Res.* (2017) 37:3175–81. 10.21873/anticanres.11677 28551661

[27] Zhang GM Zhu Y Luo L Wan FN Zhu YP Sun LJ Preoperative lymphocyte-monocyte and platelet-lymphocyte ratios as predictors of overall survival in patients with bladder cancer undergoing radical cystectomy. *Tumour Biol.* (2015) 36:8537–43. 10.1007/s13277-015-3613-x 26032095

[28] Jurasz P AlonsoEscolano D Radomski MW. Platelet-cancer interactions: mechanisms and pharmacology of tumour cell-induced platelet aggregation. *Br J Pharmacol.* (2004) 143:819–26. 10.1038/sj.bjp.0706013 15492016 PMC1575943

[29] Chen XL Xue L Wang W Chen HN Zhang WH Liu K Prognostic significance of the combination of preoperative hemoglobin, albumin, lymphocyte and platelet in patients with gastric carcinoma: a retrospective cohort study. *Oncotarget.* (2015) 6:41370–82. 10.18632/oncotarget.5629 26497995 PMC4747412

[30] Cong L Hu L. The value of the combination of hemoglobin, albumin, lymphocyte and platelet in predicting platinum-based chemoradiotherapy response in male patients with esophageal squamous cell carcinoma. *Int Immunopharmacol.* (2017) 46:75–9. 10.1016/j.intimp.2017.02.027 28268208

[31] Peng D Zhang CJ Tang Q Zhang L Yang KW Yu XT Prognostic significance of the combination of preoperative hemoglobin and albumin levels and lymphocyte and platelet counts (HALP) in patients with renal cell carcinoma after nephrectomy. *BMC Urol.* (2018) 18:20. 10.1186/s12894-018-0333-8 29544476 PMC5855974

[32] Shen XB Zhang YX Wang W Pan YY. The hemoglobin, albumin, lymphocyte, and platelet (HALP) score in patients with small cell lung cancer before first-line treatment with etoposide and progression-free survival. *Med Sci Monit.* (2019) 25:5630–9. 10.12659/MSM.917968 31356586 PMC6685331

[33] Lidoriki I Schizas D Frountzas M Machairas N Prodromidou A Kapelouzou A GNRI as a prognostic factor for outcomes in cancer patients: a systematic review of the literature. *Nutr Cancer.* (2021) 73:391–403. 10.1080/01635581.2020.1756350 32321298

[34] Yang R Cheung MC Pedroso FE Byrne MM Koniaris LG Zimmers TA. Obesity and weight loss at presentation of lung cancer are associated with opposite effects on survival. *J Surg Res.* (2011) 170:e75–83. 10.1016/j.jss.2011.04.061 21704331 PMC3154461

[35] Attaran S McShane J Whittle I Poullis M Shackcloth M. A propensity-matched comparison of survival after lung resection in patients with a high versus low body mass index. *Eur J Cardiothorac Surg.* (2012) 42:653–8. 10.1093/ejcts/ezs135 22518036

[36] Gu W Zhang G Sun L Ma Q Cheng Y Zhang H Nutritional screening is strongly associated with overall survival in patients treated with targeted agents for metastatic renal cell carcinoma. *J Cachexia Sarcopenia Muscle.* (2015) 6:222–30. 10.1002/jcsm.12025 26401468 PMC4575553

[37] Bo Y Wang K Liu Y You J Cui H Zhu Y The geriatric nutritional risk index predicts survival in elderly esophageal squamous cell carcinoma patients with radiotherapy. *PLoS One.* (2016) 11:e0155903. 10.1371/journal.pone.0155903 27196126 PMC4873221

[38] Peng SM Yu N Ren JJ Xu JY Chen GC Yang JR The geriatric nutritional risk index as a prognostic factor in patients with advanced non-small-cell lung cancer. *Nutr Cancer.* (2021) 73:2832–41. 10.1080/01635581.2020.1865423 33356605

[39] Matsuura S Morikawa K Ito Y Kubota T Ichijo K Mochizuki E The geriatric nutritional risk index and prognostic nutritional index predict the overall survival of advanced non-small cell lung cancer patients. *Nutr Cancer.* (2021) 25:1–8. 10.1080/01635581.2021.1960387 34431441

[40] Cortellini A Di Maio M Nigro O Leonetti A Cortinovis DL Aerts JG Differential influence of antibiotic therapy and other medications on oncological outcomes of patients with non-small cell lung cancer treated with first-line pembrolizumab versus cytotoxic chemotherapy. *J Immunother Cancer.* (2021) 9:e002421. 10.1136/jitc-2021-002421 33827906 PMC8031700

